# Power supply and ultrasound functionality in Malawi: findings from the 2019 harmonised health facility assessment

**DOI:** 10.1038/s44401-025-00055-y

**Published:** 2026-01-09

**Authors:** Luyanda Ngongoma, Aryn Xing, Jingning Wang, Claire R. Zhang, Pakwanja Twea, Jonathan Chiwanda, Bryan J. Weiner, Yanfang Su

**Affiliations:** 1 Department of Global Health, School of Public Health, University of Washington, Seattle, WA USA; 2 Department of Political Science, Emory University, Atlanta, GA USA; 3 International School, Bellevue, WA USA; 4 Bergen Centre for Ethics and Priority Setting, University of Bergen, Bergen, Norway; 5 Department of Planning and Policy Development, Ministry of Health, Lilongwe, Malawi; 6 Department of Clinical Services, Ministry of Health, Lilongwe, Malawi; 7 Evans School of Public Policy and Governance, University of Washington, Seattle, WA USA

**Keywords:** Health policy, Health services

## Abstract

Ultrasound sonography is a critical diagnostic tool, yet access remains limited in Malawi. This study assessed the relationship between electricity supply and ultrasound availability and functionality using data from 596 health facilities in the 2019 Harmonised Health Facility Assessment. Only 9.9% of facilities had ultrasound equipment available, with 93% of that equipment functional. Facilities powered by non-grid sources were 86% less likely to be functional (PR: 0.14; 95% CI: 0.02–0.49) compared to those with a stable grid supply. Ultrasound functionality was particularly low in primary-level facilities and those owned by the government. Spatial analysis revealed that most facilities without ultrasound were located in energy-constrained areas. While electricity access is necessary, it is insufficient on its own. Results support the need for stronger alignment between the health and energy sectors, as well as the potential for targeted deployment of handheld ultrasound in low-resource, off-grid settings.

## Introduction

Ultrasound sonography is a non-ionising, non-invasive imaging modality with diverse applications across various domains, including general, emergency, and maternal medicine^1–4^. A portable ultrasound system is defined by its ability to be easily transported and operated in various settings^5^. These systems vary in form: some are compact laptop units with dedicated ports for probe connection, while others, termed handheld devices, integrate the probe with an external screen for image display (e.g. smartphone or tablet)^6^. Together, these portable versions enable healthcare workers to conduct ultrasounds at the patient’s bedside, a practice known as point-of-care ultrasound^7^. The benefits of ultrasonography for health outcomes are well-documented in both high-income and low- and middle-income country (LMIC) health systems^4,8,9^. In clinical care, ultrasound has been shown to influence decision-making in up to 70% of cases, with one Malawi-based study reporting a ‘5-fold increase in the clinician’s decision to initiate TB treatment’^9–11^. Another example includes the promotion of maternal and neonatal health through early antenatal screening (as mandated by the World Health Organisation) and modern family planning, specifically the insertion of intrauterine devices^12^. Moreover, handheld ultrasound sonography (HHUS) has been associated with improved diagnostic confidence, patient satisfaction, and reduced pressure on radiology units through efficient task-shifting. In Malawi, where the fertility rate is 3.5 births per woman and leading causes of death include infectious diseases, neonatal conditions, and non-communicable diseases, HHUS offers a potential solution to help close diagnostic gaps within an overstretched health system^4,13,14^.

The country’s National Radiology Policy (2020–2025) emphasises the need to scale imaging capacity and address infrastructure gaps^15^. In alignment, Malawi’s Ministry of Health has advanced its digital health agenda through a National Digital Health Strategy (2020–2025) to improve equitable access to care^16^. Together, these policy frameworks advance the country’s goals under Sustainable Development Goal (SDG) 3^15,16^. The Digital Health Strategy (DHS) positions technology as a way to reduce geographical and workforce-related barriers, and in 2023, a national taskforce was formed to ‘investigate state-of-the-art information technologies and security practices, including handheld digital healthcare devices’^16,17^. HHUS aligns closely with this aim, offering advantages such as portability, adaptability to infrastructure limitations, affordability, and proven user-friendliness in both clinical and medical education settings^1,8,18^. The task force identified the ASUS LU800 handheld ultrasound as a preferred device and signalled an intent to connect it to the country’s Integrated Community Health Information System (iCHIS), thereby embedding HHUS into Malawi’s broader digital infrastructure^16,17^. Complementary efforts, largely led by NGOs, include the government-endorsed Centre of Excellence in Ultrasound at Kamuzu Central Hospital, which was announced in 2022 but remains pending construction^19,20^. Another example is the Butterfly iQ feasibility study (2021–2022), the most extensive registered HHUS implementation science study in Malawi to date, although its findings have not yet been published^21,22^. Overall, the country has articulated specific targets to ‘increase access to health services and diagnostics using digital interventions’^16^.

This momentum is supported by broader system-level investments beyond the health sector, particularly in infrastructure and energy, which are crucial for scaling diagnostic innovations, such as traditional ultrasound^23,24^. According to Weiner’s^25^ Organisational Readiness for Change (OR4C) framework, the successful adoption of health innovations (such as the integration of ultrasound into routine care) requires a strong foundation of human, financial, informational, and material resources. The latest and earlier iterations of the DHS identified connectivity, digital resources (i.e. computing devices), and power supply as three key issues related to infrastructure^16,26^. Our study examines electricity (also referred to as power or energy) as a key material resource influencing ultrasound service delivery. Previous empirical studies investigating the role of electricity in health facility readiness have primarily focused on devices such as incubators and light microscopes, with limited attention to ultrasound systems^27^. In Malawi, approximately 95% of the country’s electricity is generated through climate-sensitive hydropower, which contributes to a frequently overburdened national grid^28,29^. Consequently, Malawi ranks among the lowest globally in terms of household electricity access, with only 25.9% of the population having access to electricity. Of this, 11.3% is connected through the national grid and 14.6% through off-grid solutions^24^. The generation and distribution of electricity is managed by two state-owned entities: the Electricity Supply Corporation of Malawi and the Electricity Generation Company. However, poor intra-sectoral coordination has been identified as a key barrier to improved access for public institutions^24^. In response to these challenges and in preparation for the planned construction of approximately 900 rural healthcare facilities by 2030, Malawi joined the United Nations Sustainable Energy for All (SE4All) initiative in 2012, committing to achieving universal electricity access by 2030^23,24^.

This study aims to understand the relationship between electricity supply and the availability and functionality of ultrasound in Malawi’s healthcare facilities, particularly at the primary care level. Utilising data from the 2019 Harmonised Health Facility Assessment (HHFA), the study assesses facility-level electricity access and its implications for ultrasound deployment. By analysing these patterns, this research seeks to inform policy development and guide strategic planning around equipment procurement (traditional versus handheld ultrasound) in Malawi’s health sector.

## Results

### Descriptive statistics

Table 1 summarises the characteristics of the 596 health facilities included in the final analytic subset. Most facilities (91.10%) were primary-level, while only 8.90% were categorised as secondary or tertiary level. Government ownership was predominant, accounting for 70.64% of facilities, followed by CHAM (24.50%) and other owners 4.87%. A majority of facilities (87.08%) were located in rural areas, with the South having the greatest share (43.29%). In terms of energy supply, 24.66% of facilities had access to stable grid electricity, 46.14% were connected to an unstable grid, and 29.20% relied on non-grid sources. An analysis of outcomes showed that ultrasound is more commonly available in urban settings, CHAM-owned facilities, secondary and tertiary-level facilities, and those with a stable grid power supply. More specifically, 40.36% of urban facilities have ultrasound available compared to 5.39% in rural areas. Access is also limited at the primary level, where only 2.76% of facilities have availability compared to 83.02% among secondary and tertiary facilities. CHAM-owned facilities and those with stable grid supply performed best within their respective categories, with ultrasound availability reported at 19.18% and 23.81%, respectively. Functionality was assessed among the 59 facilities with ultrasound available, and 93% of them had functional equipment. The patterns of functionality closely follow those seen in availability. Urban facilities show higher functionality at 35.06% compared to 5.39% in rural areas. At the secondary and tertiary levels, 75.47% of facilities achieved functionality and had functional ultrasound, while only 2.76% of primary facilities did. CHAM facilities performed consistently with 19.18% reporting functional ultrasound, followed by government facilities (6.18%). Facilities with stable grid supply had the highest proportion at 22.45% compared to their counterparts, each with less than 10% ultrasound functionality. Overall, trends suggest that once a facility has ultrasound available, it is generally functional, especially when supported by stronger infrastructure.

**Table 1:** Availability and functionality of ultrasound equipment in healthcare facilities in Malawi in 2019

Variable	Total	Availability	Functionality
	*n* = 596 (100%)	*n* = 59 (9.9%)	*n* = 55 (9.2%)
Region			
Centre	226 (37.92%)	24 (10.62%)	24 (10.62%)
North	112 (18.79%)	13 (11.61%)	11 (9.82%)
South	258 (43.29%)	22 (8.53%)	20 (7.75%)
Urbanicity			
Rural	519 (87.08%)	28 (5.39%)	28 (5.39%)
Urban	77 (12.92%)	31 (40.26%)	27 (35.06%)
Facility level			
Primary	543 (91.10%)	15 (2.76%)	15 (2.76%)
Secondary and Tertiary	53 (8.90%)	44 (83.02%)	40 (75.47%)
Facility ownership			
CHAM	146 (24.50%)	28 (19.18%)	28 (19.18%)
Government	421 (70.64%)	30 (7.13%)	26 (6.18%)
Other	29 (4.87%)	1 (3.45%)	1 (3.45%)
Power supply: energy type			
Stable Grid	147 (24.66%)	35 (23.81%)	33 (22.45%)
Unstable Grid	275 (46.14%)	22 (8.00%)	20 (7.27%)
Non-grid	174 (29.20%)	2 (1.15%)	2 (1.15%)

Descriptive statistics across 597 facilities. Facility levels follow a national classification: the Primary level includes health centres, maternity facilities, and rural/community hospitals; the Secondary and Tertiary levels include district and central hospitals. Power supply categories include: stable grid = uninterrupted grid with backup; unstable grid = uninterrupted grid without backup, interrupted grid with backup and interrupted grid without backup; non-grid = off-grid and no electricity. Availability is defined as the physical presence of ultrasound; Functionality is defined as the confirmed working status of the device. Row-wise percentages reflect subgroup-specific prevalence.

**Fig. 1:**
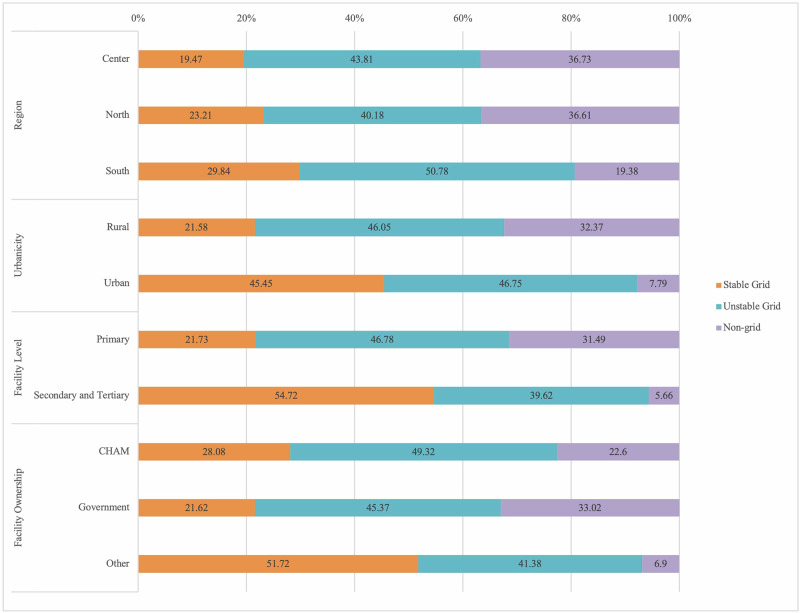
Electricity supply distribution across Malawian health facilities (2019). This figure presents grouped bar charts illustrating the distribution of electricity supply types across regions, urbanicity, facility ownership, and facility level, based on data from the 2019 Harmonised Health Facility Assessment (HHFA). The figure highlights how unstable grid supply remains dominant nationally, while stable grid access is more common in urban, secondary, and tertiary facilities, as well as those owned by CHAM. HHFA harmonised health facility assessment, CHAM Christian Health Association of Malawi.

To further contextualise the disparities observed in Table 1, we created Fig. 1 to visualise how the electricity supply types are distributed across the region, in terms of ownership, facility level, and urbanicity variables. Unstable grid supply is the most common energy type across all regions, accounting for 50.78% of facilities in the South, 43.81% in the Centre, and 40.18% in the North. Stable grid access is highest in the South (29.84%) and lowest in the Centre (19.47%), while non-grid use is relatively consistent across the regions. Urban facilities have better stable grid access at 45.45% compared to 21.58% in rural areas. Rural facilities demonstrate a greater reliance on non-grid sources, at 32.37%, compared to 7.79% in urban areas. At the facility level, 54.72% of secondary and tertiary facilities have stable grid power. Unstable and non-grid sources are more common in primary facilities at 46.78% and 31.49% respectively. Among ownership categories, CHAM facilities primarily rely on an unstable grid (49.32%), followed by stable grid (28.08%) and non-grid supply (22.60%). Government-owned facilities show lower stable grid access (21.62%) and higher reliance on unstable (45.37%) and non-grid electricity (33.02%). Facilities classified under ‘Other’ ownership that are not publicly financed reported the highest stable grid access at 51.72%.

**Fig. 2:**
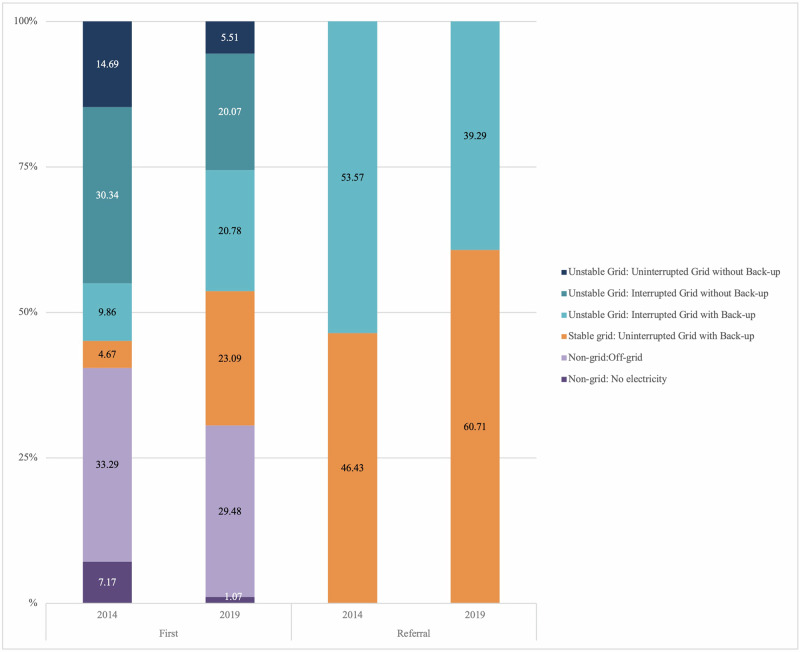
Comparison of energy type distribution by facility level between Malawi SPA (2014) and HHFA (2019). This figure compares electricity supply types across first- and referral-level facilities between two national survey periods: the 2014 service provision assessment (SPA) and the 2019 HHFA. For this visual, facility levels were classified using Suhlrie et al.’s framework: first level (health centres and rural/community hospitals) and referral level (district and central hospitals). The figure highlights improvements in uninterrupted grid access, especially at the referral level, with minimal progress in off-grid electrification. SPA service provision assessment, HHFA harmonised health facility assessment.

Using Fig. 2, we examined changes in energy supply across Malawi’s health system between 2014 and 2019. At the first level, there was a notable shift toward more reliable electricity. The proportion of facilities with uninterrupted grid access and backup increased from 4.67 to 23.09%, representing a nearly fivefold improvement. However, nearly half of first-level facilities remained on unstable grid connections. Interrupted grid access with backup rose from 9.86 to 20.78%, while those without backup declined from 30.34 to 20.07%. These shifts indicate some movement away from the least resilient supply types, but a large proportion of facilities still experience grid interruptions. Reliance on off-grid sources, such as standalone solar systems, declined only marginally, from 33.29 to 29.48%. Facilities without any electricity nearly disappeared, dropping from 7.17 to 1.07%. At the referral level, where service demands and equipment requirements are highest, stable and reliable grid access continued to improve. The share of facilities with uninterrupted grid and backup rose from 46.43 to 60.71%, while reliance on interrupted grid access decreased from 53.57 to 39.29%. No referral-level facilities reported being without electricity or using off-grid sources in either year. These findings reflect meaningful progress, particularly in electrifying and stabilising power at higher-level facilities.

**Fig. 3:**
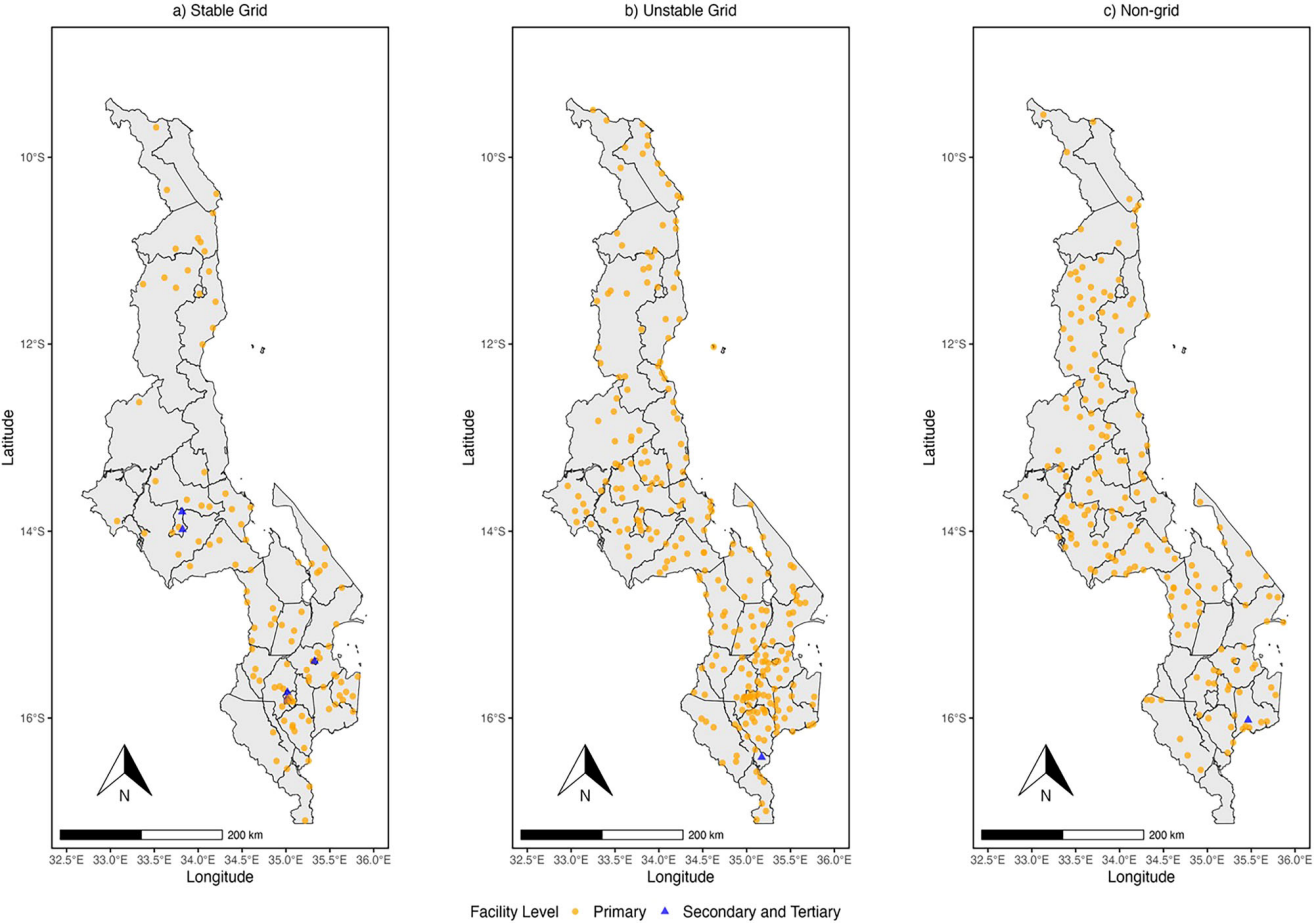
Geospatial distribution of facilities in Malawi without ultrasound, by electricity supply type and facility level (2019). This three-panel map displays the 537 health facilities without ultrasound equipment in Malawi. Facilities are stratified by electricity supply type: **a** stable grid, **b** unstable grid, and **c** non-grid. Orange circles denote primary-level facilities, while blue triangles mark secondary and tertiary facilities. The maps show that most facilities without ultrasound are primary level and rely on unstable or non-grid electricity. HHFA Harmonised Health Facility Assessment. Geospatial coordinates were visualised using geographic information system (GIS) tools in R (packages: sf, ggplot2, and ggspatial) and Map boundaries sourced from the Humanitarian Data Exchange, licensed under a Creative Commons Attribution 4.0 International License (https://creativecommons.org/licenses/by/4.0/).

Figure 3 focused on the facilities without ultrasound to identify implementation gaps and visually represent the intersection of service tier and electricity type in shaping diagnostic access across Malawi’s health system. These stratified maps also offer early insight into which facilities may be more appropriate for handheld versus traditional ultrasound technologies. Of the facilities without ultrasound (*n* = 537), the majority were primary-level (*n* = 528, 98.3%), with a few classified as secondary or tertiary-level (*n* = 9, 1.7%). Panel A shows that 112 facilities without ultrasound were operating on stable grid electricity, comprising 105 primary-level facilities and 7 secondary or tertiary facilities. These cases (concentrated in the central and southern regions) suggest that barriers to ultrasound implementation may extend beyond energy supply, including limitations in workforce capacity or equipment prioritisation. Panel B highlights the 252 facilities without ultrasound on unstable grid supply (*n* = 252), nearly all of which were at the primary level (*n* = 251). Like Fig. 3a, these facilities show a centre-south predominance. Figure 3c presents 171 facilities without ultrasound powered by non-grid energy sources. These facilities were almost primary level (*n* = 170) and geographically widespread across all three regions.

### Factors associated with ultrasound availability and functionality

Table 2 presents the crude and adjusted prevalence ratios (PRs) for the ultrasound outcomes by facility characteristics, particularly power supply type. Primary-level facilities were substantially less likely to have ultrasound available compared to secondary or tertiary-level facilities (crude PR: 0.03; 95% CI: 0.02–0.06; adjusted PR: 0.05; 95% CI: 0.03–0.11; *p* < 0.001). This association remained strong after adjustment, highlighting persistent structural disparities across facility levels. CHAM-owned facilities had significantly greater availability than government facilities (crude PR: 2.69; 95% CI: 1.60–4.51; adjusted PR: 2.13; 95% CI: 1.12–3.96; *p* = 0.019), suggesting a relative advantage in equipment distribution or management. Facilities using non-grid electricity had lower ultrasound availability (crude PR: 0.05; 95% CI: 0.01–0.16; adjusted PR: 0.15; 95% CI: 0.02–0.50; *p* = 0.009). While unstable grid supply was associated with lower ultrasound availability in unadjusted models (crude PR: 0.34; 95% CI: 0.19–0.57; *p* < 0.001), the association weakened after adjustment (adjusted PR: 0.58; 95% CI: 0.33–1.02; *p* = 0.061), indicating some partial confounding by other facility characteristics. Interestingly, rural-urban differences seen in the crude model (crude PR: 0.13; 95% CI: 0.08–0.22; *p* < 0.001) were not significant in the adjusted model (adjusted PR: 0.81; 95% CI: 0.40–1.69; *p* = 0.577). Patterns of ultrasound functionality mostly mirrored those seen in availability. Primary-level facilities were significantly less likely to report functional equipment (crude PR: 0.04; 95% CI: 0.02–0.06; adjusted PR: 0.06; 95% CI: 0.03–0.12; *p* < 0.001), which reinforces concerns about structural barriers at this level of care. CHAM facilities maintained a functional advantage over government facilities (adjusted PR: 2.31; 95% CI: 1.20–4.38; *p* = 0.011), while non-grid power supply was again significantly associated with a lower likelihood of having functional ultrasound (adjusted PR: 0.14; 95% CI: 0.02–0.49; *p* = 0.009). Facilities using unstable grid connections were also less likely to report functional ultrasound compared to those on a stable grid (adjusted PR: 0.54; 95% CI: 0.29–0.95; *p* = 0.037). Urban-rural differences in functionality, although significant in unadjusted models (crude PR: 0.15; 95% CI: 0.09–0.26; *p* < 0.001), were not statistically significant after adjustment (adjusted PR: 0.91; 95% CI: 0.44–1.91; *p* = 0.795).

**Table 2:** Crude and adjusted prevalence ratios of ultrasound outcomes and power supply energy types in Malawi

Variable	Ultrasound availability model			
	PR_crude_ (95% CI)	*p*-value	PR_adjusted_ (95% CI)	*p*-value
Region				
Centre (ref)				
South	0.8 (0.45, 1.43)	0.457	1.03 (0.56, 1.9)	0.915
North	1.09 (0.54, 2.11)	0.796	1.31 (0.63, 2.62)	0.454
Urbanicity				
Urban (ref)				
Rural	0.13 (0.08, 0.22)	<0.001	0.81 (0.4, 1.69)	0.577
Facility level				
Secondary and Tertiary (ref)				
Primary	0.03 (0.02, 0.06)	<0.001	0.05 (0.03, 0.11)	<0.001
Ownership				
Government (ref)				
Other	0.48 (0.03, 2.25)	0.475	0.18 (0.01, 0.87)	0.093
CHAM	2.69 (1.6, 4.51)	<0.001	2.13 (1.12, 3.96)	0.019
Power supply: energy type				
Stable grid (ref)				
Unstable grid	0.34 (0.19, 0.57)	<0.001	0.58 (0.33, 1.02)	0.061
Non-grid	0.05 (0.01, 0.16)	<0.001	0.15 (0.02, 0.5)	0.009

Variable	Ultrasound functionality model			
	PR_crude_ (95% CI)	*p*-value	PR_adjusted_ (95% CI)	*p*-value
Region				
Centre (ref)				
South	0.73 (0.4, 1.32)	0.299	0.95 (0.5, 1.77)	0.868
North	0.92 (0.44, 1.84)	0.83	1.1 (0.5, 2.26)	0.807
Urbanicity				
Urban (ref)				
Rural	0.15 (0.09, 0.26)	<0.001	0.91 (0.44, 1.91)	0.795
Facility level				
Secondary and Tertiary (ref)				
Primary	0.04 (0.02, 0.06)	<0.001	0.06 (0.03, 0.12)	<0.001
Ownership				
Government (ref)				
Other	0.56 (0.03, 2.62)	0.567	0.21 (0.01, 1.03)	0.129
CHAM	3.11 (1.82, 5.32)	<0.001	2.31 (1.2, 4.38)	0.011
Power supply: energy type				
Stable Grid (ref)				
Unstable Grid	0.32 (0.18, 0.56	<0.001	0.54 (0.29, 0.95)	0.037
Non-grid	0.05 (0.01, 0.17)	<0.001	0.14 (0.02, 0.49)	0.009

Results from multivariable Poisson regression models using robust standard errors to assess associations between facility characteristics and two outcomes: ultrasound availability and ultrasound functionality. Independent variables included region, urbanicity, facility level, ownership type, and electricity supply. The table presents crude and adjusted prevalence ratios, 95% confidence intervals (CI), and *p*-values.

*CHAM* Christian Health Association of Malawi, *PR* prevalence ratio, *CI* confidence interval, *Ref* reference group, *CHAM* Christian Health Association of Malawi.

### Sensitivity analysis

Results from sensitivity analyses confirmed the direction and strength of the main associations. In the backup-centric model (Table S1), facilities without backup power were 94% less likely to have ultrasound available and functional (PR = 0.06, 95% CI: 0.003–0.294; *p* = 0.006). The grid-centric model (Table S2) showed that facilities classified as non-grid were significantly less likely to have ultrasound available (PR = 0.19, 95% CI: 0.031–0.636; *p* = 0.024) and functional (PR = 0.20, 95% CI: 0.032–0.653; *p* = 0.026), respectively, compared to those connected to the grid. Associations for facility level and ownership remained robust across all model specifications.

## Discussion

This study sought to evaluate the association between power supply and ultrasound availability and functionality across 596 health facilities in Malawi. While 93% of facilities with ultrasound equipment achieved functionality, only 9.9% of all facilities had ultrasound available. Together, the findings suggest that once a facility acquires ultrasound equipment, it is generally well-maintained and functional. This contrasts with the assumption that equipment availability and functionality are equally unreliable in LMIC settings. Statistical analysis revealed considerable structural disparity across three variables: facility level, ownership, and energy type. Primary level facilities, accounting for over 90% of Malawi’s health system, were less likely to have an ultrasound available or functional, even after adjusting for other facility characteristics. These disparities mirror broader diagnostic readiness gaps identified in other national analyses. For example, Ahmed et al.^30^ found that less than 40% of primary-level facilities in Malawi were equipped to manage non-communicable diseases due to insufficient functional equipment and medicine stock, even for conditions prioritised in national plans. Additionally, ownership and electricity supply also contributed to diagnostic inequities. CHAM-owned facilities outperformed their government counterparts, and facilities relying on unstable or non-grid electricity sources were marginally disadvantaged. Consistent with the latter finding, previous research in Malawi found that facilities connected to the grid without backup, as well as those relying on off-grid supply, were significantly less likely to have functional essential diagnostics, specifically newborn incubators and microscopes^27^.

Ultrasound access in Malawi is heavily concentrated at higher levels of care. These disparities align with broader trends in LMICs, ‘where only 19% of patients access appropriate diagnostics at the primary level’^31^. In Malawi, gaps in ultrasound access at the facility level reflect long-standing health policy design choices. For example, the country’s health strategic plan does not mandate the provision of ultrasound services at maternity units^32^. This omission persists despite these facilities being responsible for delivering a Health Benefits Package (HBP), which includes services such as antenatal screening and modern family planning, where ultrasound plays a well-established role^14,33^. Historically, service inclusion within the HBP has been dependent on predetermined cost-effectiveness thresholds. Fast-evolving, more affordable technologies like HHUS represent new opportunities to expand healthcare access for Malawi’s underserved population. However, the country policy report noted that inclusion within the HBP menu does not guarantee implementation, mainly due to persistent health financing gaps^14^.

Ownership-related disparities were also evident. CHAM-managed facilities outperformed government facilities in both ultrasound availability and functionality. This may be linked to CHAM’s comparatively stronger governance structures and diversified funding sources, such as service-level agreements, donor funding and user fees^32–36^. This diversity in arrangement enables CHAMs to maintain basic service continuity, even in cases where public sector investment is limited^37^. While partnerships between the government and CHAM can help expand ultrasound access nationwide, they also present potential risks. Without clearly defined accountability mechanisms and effective coordination, there is a risk that over-reliance on CHAM may inadvertently delay the procurement of ultrasound innovations for government facilities. Over time, this could undermine the development of sustainable diagnostic capacity within the public sector and place additional strain on an already fragile partnership between the two entities^37^. Strengthening partnerships through transparency, aligned incentives, and shared responsibility will be critical to achieving sustainable basic diagnostic access at the primary level^37^.

Our evidence showed that electricity supply shaped patterns of ultrasound availability and functionality, but did not act alone. Stable grid access with backup was an essential enabler across facilities. However, Fig. 2 showed that, despite some progress in grid performance between 2014 and 2019 (particularly among referral-level facilities), nearly half of the first-level facilities continued to operate under unstable or non-grid conditions. These patterns highlight broader system gaps. While Malawi’s national energy policy targets an increase in the share of renewable energy to 96.1% by 2030, our findings at the health facility level suggest that improvements during the 2014–2019 period were concentrated on stabilising existing grid systems^24^. Regardless, planned transitions toward renewables demand even further investment in innovative energy solutions, as popular options like standalone solar systems often lack the capacity to manage medical equipment demands^24^. Our findings emphasise that electricity itself is shaped by enabling factors. Literature identifies ‘laws, policies, regulations, markets and institutions to support equitable access’^38^ as critical to the adequate provision of power supply, many of the same contextual factors that govern ultrasound deployment. Thus, suggesting that while electricity is necessary, it remains insufficient. Figure 3 reinforced this point by showing that several facilities with stable grid power still lacked ultrasound services. Nonetheless, stable electricity provides a critical foundation for community health systems^38^. The WHO identifies electricity as playing a catalytic role in strengthening system readiness^38^. Facilities with consistent energy access are better positioned to attract and retain health workers, support innovation (e.g., tele-health technologies), and expand access to universal healthcare^39,40^. These functions collectively deliver the broader contextual factors needed to support basic diagnostic imaging, particularly in rural and underserved settings^38^. Within this context, the exclusion of energy-health collaboration in Malawi’s 2020 National Radiology Policy^15^ signals a critical gap. Aligning diagnostic expansion with energy-sector development (particularly through strengthened cross-ministerial collaboration) represents a significant opportunity to advance diagnostic equity. Better integration between energy and health planning will be essential to ensure that electricity investments, including renewable innovations, meaningfully improve diagnostic readiness at all levels of care.

Figure 3 mapped the distribution of facilities without ultrasound, categorised by electricity type and facility level. While we do not recommend that all such facilities automatically receive equipment, the map provides a valuable geographic profile to inform strategic expansion. It highlights where diagnostic gaps are most concentrated and can help guide decisions about which areas may benefit from ultrasound investment and what device type is most appropriate, depending on electricity access and facility level. Facilities with stable grid supply but no ultrasound equipment (Panel A) were mostly at the primary level. In select cases, these may be suitable candidates for conventional cart-based systems, provided consistent and sufficient power is available. Beyond energy compatibility, both types of ultrasound will require supporting systems. These include equipment maintenance routines and the use of evidence-based clinical guidelines^4,18^. Ongoing implementation initiatives (e.g. Centre of Excellence in Ultrasound) will be key to supporting this transition. When matched to the local energy environment and supported by adequate system infrastructure, HHUS offers a promising pathway to expand diagnostic access in underserved areas.

This study offers several strengths. It utilises nationally representative data from the country’s HHFA, allowing for broad generalisability across Malawi’s public and non-public health sectors. While the HHFA data used in this study are from 2019, they remain the most recent and high-quality nationally representative dataset for Malawi^35,39^. Moreover, electricity access in sub-Saharan Africa continues to progress slowly, making the findings contextually relevant despite the data’s age^41^. The inclusion of stratified spatial maps (Fig. 3) enhances contextual interpretation by linking facility characteristics to regional electricity profiles. Adjusted regression models were applied to control for confounding across key facility variables, improving the internal validity of our findings. Sensitivity analyses were conducted using two alternative electricity groupings, with consistent direction and strength of associations observed across both approaches, reinforcing the stability of our results. Additionally, direct linkages were made between facility-level energy profiles and national policy priorities, strengthening the study’s relevance for diagnostic planning and energy investment strategies. While grounded in Malawi’s system, the approach and findings are likely relevant to other sub-Saharan African settings undergoing similar transitions in infrastructure and primary care expansion. However, there are important limitations to acknowledge. The cross-sectional design limits causal inferences between the type of electricity and ultrasound outcomes. Electricity supply was recorded categorically and did not differentiate between specific sources (e.g. solar, generator, or battery), which restricted interpretation, especially regarding progress in renewable energy. Ultrasound availability and functionality were assessed based on a single day’s observation, which may not capture longer-term patterns. Lastly, this study focused solely on material resources, specifically electricity, as outlined in the (OR4C) framework. Other foundational OR4C elements, such as human, financial, and informational resources, were not assessed and remain important areas for future exploration.

This study examined the role of electricity supply in shaping the availability and functionality of ultrasound services across Malawi’s health facilities. Findings showed that stable grid access improves ultrasound readiness, yet it remains insufficient without broader system support. Disparities at the facility level and by ownership point to structural design gaps that extend beyond energy access alone. As Malawi expands its diagnostic capacity, handheld ultrasound offers a potentially cost-effective and energy-efficient solution for future integration into HBPs, particularly at the primary level and in energy-constrained settings. Mapping facility characteristics alongside electricity profiles, as demonstrated here, can guide energy-informed procurement and strategic ultrasound planning. Broader intra-sectoral coordination within ministries is needed to facilitate inter-sectoral collaboration between the energy and health sectors. This foundation can support strengthened entity partnership between CHAM and government actors, improve policy alignment, and contribute to more integrated energy-infrastructure design and renewable investment.

## Methods

### Study design and setting

A cross-sectional study design was employed, using survey findings from the 2019 Malawi HHFA facility inventory, which was conducted between November 2018 and March 2019^30,42^. Facilities included in the HHFA survey were identified using the Malawi Facility List (MFL)^30,42^. The MFL identifies 1224 facilities and provides a comprehensive overview of the country’s healthcare facilities. The Malawi HHFA used a census strategy to survey facilities from the MFL. HHFA successfully captured data from 1106 of the total 1224 facilities, as 118 facilities were ‘either not located or no longer existed’^30,42^. Data were reviewed by the Global Fund as part of quality oversight support^35^.

### Sampling and selection

Ultrasound is listed in the World Health Organisation’s (WHO) priority medical device guidance as an essential device at the health centre level and above^43^. This secondary analysis restricted the HHFA dataset to all 596 facilities that offered ultrasound services as per national guidelines in the census health facility survey. These eligibility criteria were guided by the Malawi Health Sector Strategic Plan II (2017–2022) (HSSP II) and supporting literature, which identify health centres, rural communities, districts, and central hospitals as suitable sites for ultrasound services^30,32^. Primary care facilities include health centres and rural community hospitals; district hospitals represent the secondary level, while central hospitals constitute the tertiary level^33,44^. Primary maternity facilities were also included as their service scope indicated potential suitability for ultrasound, such as routine early pregnancy screening (≤24 weeks gestation) and intrauterine device insertion^45^. Excluded were health posts, dispensaries, clinics and mental health hospitals in this study. Regarding ownership, two types of publicly financed facilities were included: government-owned and facilities operated by the Christian Health Association of Malawi (CHAM). Facilities not classified as government or CHAM were grouped as ‘other’. The final subset for this secondary analysis comprised 543 primary-level facilities, 47 secondary facilities, and 6 tertiary-level facilities. This study is a secondary analysis of facility data from the 2019 Malawi HHFA and is not determined as human subject research, such that IRB approval is not required.

### Data and variables

Electricity supply serves as the primary independent variable in this study. This variable was constructed using three HHFA survey questions related to: the facility’s main power source (national grid or off-grid), the reliability of the grid (defined as having no interruptions longer than two hours during operating hours in the past seven days) and the presence of a backup power source^46^. This approach to the construction of the variable was adapted from Suhlrie et al.^27^ empirical analysis of Malawi’s 2014 Service Provision Assessment(SPA) and used similar survey questions to classify the facilities into six electricity categories: uninterrupted grid with backup, interrupted grid with backup, uninterrupted grid without backup, interrupted grid without backup, off-grid electricity, and no electricity. In our study, these six types were consolidated into three categories to facilitate statistical analysis. This decision was informed by the absence of facilities classified as either ‘uninterrupted grid without backup’ or ‘no electricity’ across the two ultrasound outcomes. Our classification was therefore as follows:

- Stable grid: Facilities with uninterrupted grid power and a backup power source.
- Unstable grid: Facilities are grid-supplied but face challenges such as frequent interruptions or lack a backup power source (interrupted grid with backup, interrupted grid without backup).
- Non-grid: Facilities relying on off-grid electricity as the main power source (solar, battery, fuel-based generator, and hybrid).

Other key facility characteristics included urbanicity (urban vs. rural), facility level (primary, secondary or tertiary), facility ownership (government, CHAM, or other), and region (North, Centre or South). The dependent variables were derived from a single HHFA equipment inventory question, which asked field teams: ‘Please tell me if ultrasound equipment is available and functional today?’ Response options included: available and functional; available, not functional; available, do not know if functional; and not available. For this analysis, these responses were disaggregated into two binary outcomes: availability (yes or no) and functionality (yes or no). Availability was defined as any facility where ultrasound was observed or reported to be present, while functionality referred to ultrasound being observed and confirmed to be working. As the data did not specify the type, availability, or functionality of the ultrasound, they can refer to any type of ultrasound. To our knowledge, cart-based ultrasound was likely referred to in the HHFA, as the national HHUS rollout has only recently entered discussion in 2023, and the most extensive feasibility study concluded in 2024^16,21^.

### Statistical analyses

The statistical analysis examined the relationship between electricity supply and the availability and functionality of ultrasound equipment in Malawian health facilities. Findings were intended to explore the potential suitability of HHUS in energy-constrained Malawi. Descriptive statistics were used to summarise the ultrasound outcomes across the key variables: region, urbanicity, facility level, ownership, and electricity supply. Thereafter, electricity supply was described across these same key variables to provide contextual insight into the energy landscape. To examine changes in electricity supply over time, we compared our findings from the 2019 HHFA with those from Suhlrie et al.^27^ 2014 analysis. The HHFA is an extension of the SPA’s core measurements, thus allowing for valid comparisons across survey periods^44^. To facilitate direct comparison, we briefly adopted their categorisation of facilities into two levels: first-level facilities (health centres, community hospitals, and other rural hospitals) and referral-level facilities (district and central hospitals). To conduct association analysis, adjusted Poisson regression (95% CI) with robust standard errors was used to model the associations between electricity type and the two binary outcomes, ultrasound availability and ultrasound functionality. The choice of Poisson regression over logistic regression was informed by literature that recommends PRs over odds ratios in cross-sectional studies to avoid overestimation^47–49^. Outcomes were tested separately from each other, with identical covariates: region, urbanicity, facility level, ownership, and electricity type. The final model is specified in Eq. (1):

$$log\,log\,it\,\left(\gamma j\right)={\beta }_{0}+{\beta }_{1}{Energytypej}+{\beta }_{2}{Urbanicityj}+{\beta }_{3}{FacilityLevelj}+{\beta }_{4}{FacilityOwnershipj}+{\beta }_{5}{Regionj}+\varepsilon j$$

These variables were selected based on their theoretical relevance and the support they have in the literature. Analyses were performed using R Studio Version 2024.09.0 + 375^27^. In this model, $\gamma j$ represents the binary outcome (1 = ultrasound is available or functional; 0 = ultrasound is not available or not functional) for the facility. The covariates include ${\beta }_{1}$ energy type, ${\beta }_{2}$ for urbanicity, ${\beta }_{3}$ for facility level, ${\beta }_{4}$ for facility ownership, and ${\beta }_{5}$ for region. To assess the robustness of our findings to the operationalisation of electricity supply, we conducted sensitivity analyses using two alternative categorisations of the energy supply variable. First, a backup-centric classification grouped facilities based on whether they had access to any backup power source (regardless of their primary energy type), creating two categories: Backup Power and No Backup Power. In this test, off-grid facilities were also further disaggregated based on whether they reported a functioning secondary source of electricity. Facilities were categorised as Backup Power if they had uninterrupted or interrupted grid with backup, or off-grid electricity with backup; and as No Backup Power if they had grid without backup, off-grid without backup, or no electricity. This refined grouping tested whether power redundancy alone was associated with ultrasound availability and functionality. Second, we applied a grid-centric classification that grouped facilities into grid-connected and non-grid-connected. Grid-connected facilities included all forms of national grid supply, whether uninterrupted or interrupted, and with or without backup. Non-grid facilities relied on off-grid electricity or had no power supply, without further disaggregation by backup status. For all sensitivity models, facility level, ownership, region, and urbanicity were controlled for. Complete model outputs are provided in Supplementary Tables S1 and S2.

Traditional ultrasound systems typically require 800–1000 watts and a steady alternating current supply^38^. In contrast, many facilities in Malawi operate on unstable or non-grid electricity sources, which present infrastructure constraints. In these settings, HHUS offers a more adaptable solution. Handheld devices are portable, battery-powered, and require only 6–28 W, making them suitable for most primary health centres in Malawi, where the daily electricity supply averages just 8.2 and 1.9 kW-h^38,50^. Based on these technical specifications and global guidance, HHUS may be more appropriate for facilities with non-grid or unstable electricity, while traditional cart-based ultrasound systems may be better supported in stable-grid environments. We generated a three-panel map using spatial coordinates from the HHFA to identify implementation gaps and overlaps between electricity type and service tier, focusing on facilities that reported no ultrasound availability (*n* = 537). Facilities missing latitude or longitude data were excluded (*n* = 2). The facilities in the map were stratified by facility level and electricity type: Panel A (stable Grid), Panel B (unstable Grid), and Panel C (non-grid). Shapefiles outlining national and district boundaries were obtained from the Humanitarian Data Exchange and loaded using the sf package in R^51^. Each subset of facilities was converted into a spatial object and mapped using the ggplot2, ggspatial, and ggpubr packages^52–54^.

## Supplementary information

Supplementary Information

## Data Availability

The data supporting the findings of this study are available from the Malawi Ministry of Health; however, restrictions apply to the availability of these data, which were used under licence for the current study and are therefore not publicly available. Data are available from the authors upon reasonable request, provided that permission is obtained from the Ministry of Health in Malawi.

## Code availability

The code used in this study is not publicly available but was provided to the journal and peer reviewers for evaluation. It can be obtained from the corresponding author upon reasonable request.
