# Supplementary material for: Power supply and ultrasound functionality in Malawi: findings from the 2019 harmonised health facility assessment
Source: Npj Health Syst. 2026 Jan 9;3:1. doi: 10.1038/s44401-025-00055-y (PMC13354168; doi:10.1038/s44401-025-00055-y)
Supplement: Supplementary file 1 — Supplementary Information [file 44401_2025_55_MOESM1_ESM.docx]

# **SUPPLEMENTARY**

**Table Legend**

| Table S1. Sensitivity analysis of adjusted prevalence ratios (PRs) for ultrasound outcomes by backup power classification, Malawi 2019 HHFA | Multivariable Poisson regression models with robust standard errors were used to estimate PRs and 95% confidence intervals (CIs), comparing facilities with backup power (interrupted grid with backup, uninterrupted grid with backup, or off-grid with backup) to facilities without backup power (interrupted grid without backup or off-grid without backup). Models were adjusted for region, urbanicity, facility level, and ownership. PR = Prevalence Ratio; CI = Confidence Interval; Ref = reference group |
| --- | --- |
| Table S2. Sensitivity analysis of adjusted prevalence ratios (PRs) for ultrasound availability and functionality by grid connection status, Malawi 2019 HHFA | Multivariable Poisson regression models with robust standard errors were used to estimate PRs and 95% confidence intervals (CIs), comparing grid-connected facilities to non-grid facilities (solar, battery, fuel generator, or hybrid systems). Models were adjusted for region, urbanicity, facility level, and ownership. PR = Prevalence Ratio; CI = Confidence Interval; Ref = reference group |

| Table S1. Sensitivity analysis of adjusted prevalence ratios for ultrasound outcomes by backup power classification, Malawi 2019 HHFA | | |
| --- | --- | --- |
| **Variable** | **Ultrasound availability** | |
|  | **PR**_adjusted_ **(95% CI)** | **p-value** |
| **Power Supply: Energy Type** |  |  |
| Backup Power (ref) |  | |
| No Backup Power | 0.06 (0.003, 0.294) | 0.006 |
| **Region** |  |  |
| Center (ref) |  | |
| South | 0.91 (0.502, 1.655) | 0.766 |
| North | 1.27 (0.617, 2.518) | 0.498 |
| **Urbanicity** |  |  |
| Urban (ref) |  | |
| Rural | 0.74 (0.363, 1.527) | 0.408 |
| **Facility Level** |  |  |
| Secondary and Tertiary (ref) |  | |
| Primary | 0.09 (0.039, 0.178) | <0.001 |
| **Ownership** |  |  |
| Government (ref) |  | |
| Other | 0.21 (0.012, 1.036) | 0.133 |
| CHAM | 1.86 (0.977, 3.449) | 0.052 |
| **Variable** | Ultrasound Functionality | |
|  | **PR**_adjusted_ **(95% CI)** | **p-value** |
| **Power Supply: Energy Type** |  | |
| Backup Power (ref) |  | |
| No Backup Power | 0.06 (0.003, 0.292) | 0.006 |
| **Region** |  |  |
| Center (ref) |  | |
| South | 0.83 (0.446, 1.520) | 0.545 |
| North | 1.08 (0.501, 2.211) | 0.831 |
| **Urbanicity** |  |  |
| Urban (ref) |  | |
| Rural | 0.80 (0.389, 1.682) | 0.554 |
| **Facility Level** |  |  |
| Secondary and Tertiary (ref) |  | |
| Primary | 0.09 (0.042, 0.192) | <0.001 |
| **Ownership** |  |  |
| Government (ref) |  | |
| Other | 0.26 (0.014, 1.258) | 0.186 |
| CHAM | 2.04 (1.058, 3.860) | 0.029 |
| * PR = Prevalence Ratio, CI = confidence Interval, Ref = reference group | |  |

| Table S2. Sensitivity analysis of adjusted prevalence ratios for ultrasound availability and functionality by grid connection status, Malawi 2019 HHFA | | |
| --- | --- | --- |
| **Variable** | Ultrasound Availability | |
|  | **PR**_adjusted_ **(95% CI)** | **p-value** |
| **Power Supply: Energy Type** |  |  |
| Grid-connected (ref) |  | |
| Non-grid | 0.19 (0.031, 0.636) | 0.024 |
| **Region** |  |  |
| Center (ref) |  | |
| South | 0.97 (0.534, 1.769) | 0.933 |
| North | 1.43 (0.695, 2.839) | 0.313 |
| **Urbanicity** |  |  |
| Urban (ref) |  | |
| Rural | 0.75 (0.368, 1.548) | 0.428 |
| **Facility Level** |  |  |
| Secondary and Tertiary (ref) |  | |
| Primary | 0.05 (0.024, 0.105) | <0.001 |
| **Ownership** |  |  |
| Government (ref) |  | |
| Other | 0.21 (0.011, 1.011) | 0.126 |
| CHAM | 2.11 (1.109, 3.930) | 0.02 |
| **Variable** | Ultrasound Functionality | |
|  | **PR**_adjusted_ **(95% CI)** | **p-value** |
| **Power Supply: Energy Type** |  |  |
| Grid-connected (ref) |  | |
| Non-grid | 0.20 (0.032, 0.653) | 0.026 |
| **Region** |  |  |
| Center (ref) |  | |
| South | 0.88 (0.474, 1.619) | 0.684 |
| North | 1.22 (0.565, 2.498) | 0.592 |
| **Urbanicity** |  |  |
| Urban (ref) |  | |
| Rural | 0.82 (0.397, 1.710) | 0.583 |
| **Facility Level** |  |  |
| Secondary and Tertiary (ref) |  | |
| Primary | 0.05 (0.025, 0.113) | <0.001 |
| **Ownership** |  |  |
| Government (ref) |  | |
| Other | 0.25 (0.014, 1.235) | 0.18 |
| CHAM | 2.31 (1.196, 4.370) | 0.011 |
|  |  |  |
| * PR = Prevalence Ratio, CI = confidence Interval, Ref = reference group | | |
